# Pulmonary Function in Paediatric Patients with Inflammatory Bowel Disease

**DOI:** 10.3390/jcm11206095

**Published:** 2022-10-16

**Authors:** Katarzyna Bąk-Drabik, Michalina Malik, Karolina Gwoździewicz, Anna Jarzumbek, Helena Krakowczyk, Monika Głowinkowska, Piotr Adamczyk

**Affiliations:** 1 Department of Paediatrics, Faculty of Medical Sciences in Zabrze, Medical University of Silesia, 40-055 Katowice, Poland; 2 Clinical Hospital No 1, 41-800 Zabrze, Poland; 3 Department of Pneumonology and Cystic Fibrosis, Institute of Tuberculosis and Pulmonary Diseases, 34-700 Rabka Zdrój, Poland; 4 Department of Paediatrics, Faculty of Medical Sciences in Katowice, Medical University of Silesia, 40-055 Katowice, Poland

**Keywords:** pulmonary function, inflammatory bowel disease, children

## Abstract

Background: Among the extraintestinal manifestations of inflammatory bowel disease (IBD), those involving the lungs are relatively rare and often overlooked. There are only scarce data on the prevalence of IBD-associated lung involvement in children. Objectives: The aim of our study was to assess pulmonary function in IBD children by different methods and to evaluate the influence of immunosuppressive therapy on disease severity. Methods: Seventy-two children with IBD (mean age of 14.45 ± 2.27 years) and 40 age-matched healthy controls (mean age of 14.17 ± 2.82) were included in the study. Pulmonary function tests (PFTs) were carried out by means of spirometry, oscillometry (IOS) and fractional exhaled nitric oxide (FeNO) to assess the pulmonary involvement. Results: Certain differences were observed between the study group and the control group, regarding the spirometric and oscillometry parameters. The fractions of exhaled nitric oxide did not differ between the group with IBD patients and the control group with regards to disease activity, the duration of illness and the administered immunosuppressive treatment. Conclusions: The mean spirometry results were significantly different in the study group compared to the controls, although they were still within the normal limits. The pulmonary function abnormalities did not depend on either the disease activity or the immunosuppressive therapy. Oscillometry could be a supplementary method to assess pulmonary resistance. In turn, FeNO does not appear to be useful either in screening IBD children for pulmonary involvement or for the evaluation of disease activity. It appears then that only general screening of asymptomatic patients is a suitable method and a necessary recommendation in this population, prompting a revision of the current diagnostic approach.

## 1. Introduction

Inflammatory bowel disease (IBD) is a chronic inflammatory disorder of unknown aetiology that commonly involves the gastrointestinal tract. Crohn’s disease (CD) and ulcerative colitis (UC) are the two main forms of chronic IBD [1]. Although the mentioned diseases most often affect the gastrointestinal tract, their extraintestinal manifestations are also commonly observed, including the skin, the joints and, less often, the lungs. The common embryonic origin of both colonic and respiratory epithelia from the primitive foregut and the similarity of the mucosal immune system may be responsible for similar pathological changes. Thus an injury to the intestinal barrier due to the circulating immune complexes in IBD, may result in an entry of antigens that may induce both local and systemic inflammations [1,2,3,4]. There is a wide array of lung manifestations, ranging from subclinical alterations, via airway and parenchymal lung disease, to pleural and drug-related conditions [1,4].

Many reports describe pulmonary involvement in adult patients with IBD [5,6,7]. The prevalence of respiratory involvement in adults is estimated at 21–40%, but our knowledge about the pulmonary involvement in paediatric IBD is rather scarce, while its exact prevalence is really unknown [1]. El Amrousy et al. reported subclinical pulmonary function test (PFTs) abnormalities as common in children with IBD even during the remission period, concluding that periodic PFT evaluation should be considered a part of the routine follow-up of IBD children [8]. On the other hand, a Polish study revealed a low prevalence of pulmonary involvement in IBD children [9]. An early diagnosis of pulmonary involvement in IBD children seems, thus, to be very important, as an early correct diagnosis would provide reliable opportunities for effective treatment. Most of these conditions are steroid-responsive [10]. However, The European Society for Paediatric Gastroenterology, Hepatology and Nutrition (ESPGHAN) did not consider pulmonary involvement to be a feature of paediatric IBD that warrants regular investigation [11]. 

To sum up, the possessed data are still insufficient to establish the prevalence and significance of respiratory involvement in the paediatric IBD population. 

The objectives of our study were two-fold: (1) to assess the pulmonary function in children with IBD, using different methods, and (2) to evaluate the relationships between the abnormalities in pulmonary tests and IBD activity and the applied immunosuppressive treatment. 

All subjects gave their informed consent for inclusion before they participated in the study. The study was conducted in accordance with the Declaration of Helsinki, and the protocol was approved by the Ethics Committee of the Medical University of Silesia, Katowice Approval Code: KNW/0022/KB1/37/19, Approval Date: 12 March 2019.

## 2. Material and Methods

### 2.1. Subjects

Seventy-two children (36 girls and 36 boys) with IBD, their mean age being 14.45 ± 2.27 years, treated at the Public Clinical Hospital in Zabrze during the years 2019–2021, were enrolled to the study as a study group. The inclusion criteria were as follow: the age between 6 and 18 years and the established diagnosis of IBD, based on the Porto Criteria [12]. On the other hand, the exclusion criteria were as follows: the respiratory tract infection within the preceding 3 weeks, a denial or withdrawal of consent and an inability to perform repeatable lung function measurements. The following clinical parameters were measured in each patient: the anthropometric parameters, illness duration, the activity of the disease and the methods of treatment. The disease duration was defined as the period between the date of the onset of symptoms and the date of PFTs. Ulcerative colitis (UC) and Crohn’s disease (CD) activities were determined by means of PUCAI (Pediatric Ulcerative Colitis Activity Index) and PCDAI (Pediatric Crohn’s Disease Activity Index), respectively. An active disease was defined by PCDAI score > 10 [13] and PUCAI > 10 [14]. 

The patients did not complain of any pulmonary symptoms, revealing no changes in physical examinations or reporting any previous respiratory diseases in history. 

The control group consisted of 40 children (their mean age being 14.17 ± 2.82) between the age of 6 and 17 years, all of them without any respiratory or chronic digestive tract disease. The inclusion criteria for the control group were as follows: normal growth and general development, the absence of personal or familiar history of wheezing, asthma, allergic rhinitis or eczema, no exposure to cigarette smoke at home, no history of premature birth or low birth weight, no cardiac disease, obesity or muscle disease and no malignancy or upper respiratory tract infections within the previous 3 weeks. 

There were no smokers, either in the study group or in the control group. The weight of the study group was significantly lower vs. the control group (−0.32 ± 0.95 vs. 0.35 ± 1.37, respectively). 

### 2.2. Pulmonary Function Tests (PFTs)

Pulmonary spirometry tests were carried out in both groups to assess lung ventilation and FeNO was used as the indirect marker of inflammation. Due to the lack of consent, IOS was performed in only 46 patients of the study group and in 40 patients of the control group.

#### 2.2.1. Spirometry

Spirometry assessment was performed, using a JAEGER Vyntus PNEUMO spirometer (CareFusion, Heidelberg, Germany, 2016), according to the ATS/ERS guidelines [15]. The best values of a minimum of three acceptable measurements were taken. The reported values of forced expiratory volume in one second (FEV1), forced vital capacity (FVC), FEV1/FVC and forced mid expiratory flow (MEF25-75) were recorded and juxtaposed with GLI-2012 references. 

All the pulmonary function parameters are presented as the number of standard deviations from corresponding reference values (z-score). An airway obstruction was diagnosed if z-score for FEV1/FVC ratio was below −1.64. An abnormally small airway flow was diagnosed if MEF50 and MEF25 z-scores were below −1.64.

#### 2.2.2. FeNO Measurement

FeNO was measured before spirometry by the single-breath method, using a chemiluminescence NO analyser (Medisoft Hypair FeNO, Sorrines, Belgium; Provita, Strzelce Opolskie, Poland), according to the American Thoracic Society/European Respiratory Society (ATS/ERS) guidelines [16]. The measurements were made in triplicate in 30-second intervals, and the mean values were taken as an input for analyses. FeNO values are expressed in parts per billion (ppb). The obtained results were compared with the control group. The assessment of nitric oxide (NO) concentration in exhaled air is a recognized method to monitor the airway inflammation in asthma, as it has been found that the concentration of exhaled nitric oxide is proportional to the degree of inflammation in the bronchial wall, eosinophilia in sputum and bronchial hyperresponsiveness [16]. It has been hypothesized that nitrogen pathways may contribute to the IBD-related lung disease, while its metabolites may serve as the markers of lung involvement in IBD [17,18]. The normal range of FeNO is very difficult to establish. The American Thoracic Society recommends that FeNO values above 35 ppb in children be used to indicate that eosinophilic inflammation and, in symptomatic patients, responsiveness to corticosteroids are likely. In turn, FeNO levels below 20 ppb in children should be used to indicate that eosinophilic inflammation and responsiveness to corticosteroids are less likely [19]. 

#### 2.2.3. Impulse Oscillometry (IOS)

Impulse oscillometry is a non-invasive method to measure the lung impedance, defined through the parameters of resistance (R) and reactance (X). IOS assesses the mechanical properties of the lung, while spirometry reflects the characteristics of the airflow. It has been shown that IOS parameters are more sensitive for identifying patients with asthma and for excluding those without asthma than the parameters of spirometry. In addition, IOS is useful in the follow-up of these patients and may detect airway obstructions earlier than spirometry [20]. 

One of the main indications of IOS in children results from an assessment of patients with asthma. Other indications for oscillometry include: cystic fibrosis, infection by rhinovirus, post-infectious bronchiolitis obliterans, gastroesophageal reflux disease, chronic obstructive pulmonary disease and obesity.

IOS (JAEGER Vyntus IOS, Care Fusion, Germany, 2016) was used to measure the input impedance of the respiratory system. Oscillometric measurements were performed during tidal breathing, with the patient sitting, breathing quietly, with a clip nose. Three to five sequences of breaths, lasting at least 30 s, are suitable for the analysis. 

The acceptability criteria for IOS measurements included the coherence values being higher than or equal to 0.6 at 5 Hz and higher than or equal to 0.8 at 10 Hz, the test coefficient of variation of Rrs being less than or equal to 15% (with a minimum of three tests and the absence of the following features within the flow tracing, gauged by visual inspections (swallowing, glottis closure, leaks around the mouthpiece, an improper seal with the nose clip)) [21]. In practice, the following parameters had a diagnostic importance and were chosen for analysis and interpretation (see Table 1). The percentage of predicted values and z-scores for IOS parameters were calculated, according to the reference equations [22]. Table 2 shows the main changes in the IOS parameters in different ventilatory disorders.

## 3. Statistical Analysis

A statistical analysis was performed, using the Statistica software (StatSoft, Tulsa, OK, USA). The mean values and standard deviations were used for descriptive statistics of continuous variables. The normality of data distribution was verified by the Shapiro–Wilk test. Absolute values and percentages were provided for qualitative variables.

The Student’s *t*-test for independent samples or the Mann–Whitney U test was applied for comparative analyses of continuous variables, depending on data distribution. Correlation analyses were carried out, using the Pearson test or the Spearman test, whichever was appropriate, according to data distribution. The significance of results in all the statistical analyses was assumed at *p* < 0.05. The Bonferroni correction was applied in case of multiple comparisons.

## 4. Results 

The characteristic features of the patients from the study group are presented in Table 3.

### 4.1. Spirometry

The spirometry results, presented as the number of standard deviations from the reference values (z-scores), as well as the mean of results, differed significantly in the IBD patients compared to the study group. However, the mean values in both groups were within their normal limits. The results are presented in Table 4. 

Nineteen (26%) IBD patients, but only one (2.5%) subject of the control group, showed abnormal spirometry results. Airway obstructions were observed in 10 (14%) IBD patients (7 with CD and 3 with UC). Airway obstructions with small abnormalities were observed in three (4.1%) (two with CD and one with UC) subjects from the study group, while in none of the subjects in the control group. A restrictive ventilatory pattern was observed in six patients (8.3%) (four with CD and two with UC) but in none of the controls. None of the patients with abnormal tests presented any symptoms from the respiratory tract.

### 4.2. FeNO Measurements

The mean FeNO levels in IBD patients did not differ from those in the control group (16.37 ± 11.46 ppb vs. 18.15 ± 12.71 ppb, respectively) (*p* = 0.45) and ranged from 5 to 80 ppb. Only three patients showed abnormal FeNO levels, exceeding the upper limit of normal (>35 ppb), similar to the control group. There were no significant differences between CD and UC patients (16.06 ± 4.26 ppb vs. 16.64 ± 8.04 ppb, respectively) (*p* = 0.83). Forty-three out of seventy-two (59%) FeNO measurements were performed under immunosuppressive or immunomodulatory treatment. There were no significant differences in FeNO measurement results between the children with or without immunosuppressive or immunomodulatory treatment (16.06 ± 8.02 ppb vs. 16.82 ± 13.27 ppb, respectively) (*p* = 0.78). There were also no significant differences depending on disease activity, based on PCDAI and PUCAI scores (*p* = 0.16) (see Table 4).

### 4.3. Impulse Oscillometry (IOS)

A significant difference was observed between X5 z-score (−0.01 ± 0.67 vs. 0.27 ± 0.63, respectively, *p* < 0.05) and the resonant frequency 16.21 ± 4.2 vs. 14.16 ± 5.24, respectively, *p* < 0.05) between the study group and the control group (see Table 4). Z -scores of R5, R20, R5–20, X20 HZ and AX did not differ in the IBD patients compared to those in the study group. In the CD group, the patients, when compared to the control group, revealed a significant difference in terms of Fres (17.13 ± 3.92 vs. 14.16 ± 5.24, respectively). It was not observed in the group of children with UC, nor did we observe any differences related to the activity of the disease, its duration or to the applied immunosuppressive therapy (see Table 5). 

Before further analyses, the CD and UC were compared, with no significant differences in the analysed parameters. For this reason, the IBD group was treated as a whole for further analyses. 

### 4.4. Differences in PFTs, Related to the use of Immunosuppressive Therapy and the Disease Activity

The results of spirometry, oscillometry and FeNO, obtained in the patients under immunosuppressive therapy, did not differ from those in the patients without immunosuppressive therapy (see Table 5). Among the 19 patients with abnormal spirometry results, 14 were under immunosuppressive therapy and presented obstructive (8), obstructive and small airways (2) and a restrictive pattern (4). 

A comparison of the patients with different disease activities did not show any significant differences. In the patients with abnormal spirometry results, 13 patients presented mild activity, 2 moderate activity and 1 severe activity of the disease. 

The patients without immunosuppressive therapy were taller than those under immunosuppressive therapy (−0.47 ± 0.89 vs. 0.03 ± 1.14, respectively, *p* = 0.01) (Table 5).

### 4.5. Correlations among IOS, Spirometry and FeNO

In our study, the correlations among the z-scores of spirometric, IOS and FeNO results were evaluated. Only a few correlations were observed. 

The Z-scores of FEV1/FVC significantly negatively correlated with the z-scores of AX (*p* = 0.39), R5–20% (*p* = 0.06) and the resonant frequency (*p* = 0.41). The z-scores of FVC significantly negatively correlated with the z-scores of R20. FeNO significantly correlated with H20 z-score. The z-scores of FEV1, FVC and PEF did not correlate with any of the IOS parameters.

## 5. Discussion

### 5.1. Spirometry Results

The spirometry results, as presented in our report, differed significantly in the IBD children compared to the control group, but the mean values in both groups were within their normal limits. 

The prevalence of abnormal spirometry results was observed in 19 (26%) IBD patients but only in 1 (2.5%) subject of the control group. Airway obstructions were observed in 10 (14%) IBD patients, airway obstructions with small airway test abnormalities were observed in 3 (4.1%) patients and a restrictive ventilatory pattern was observed in 6 patients (8.3%). No one from the control group presented any obstructive, small airway or restrictive abnormalities. 

A similar observation was reported in El Amrousy’s study [8]: a small airway obstruction was observed in 19% of studied children, while a restrictive pattern of lung dysfunction was detected in 15% of the subjects. Conversely, in a Polish study concerning children with IBD, the prevalence of lung impairments was low: a restrictive ventilatory pattern was demonstrated in only 2% of the patients and an airway obstruction was identified in 4% of the study group, and the results of small airway tests were abnormal in 26% of the subjects from the study group, but the mean result did not differ from that in the control group [9]. In another paediatric study, significantly mildly decreased mid- and end-expiratory flow values (MEF25–50) in CD patients were observed when compared to the controls, while the larger airways seemed unaffected, as reflected by normal FEV_1_ and FEV_1_/VC values [23].In the Yammine et al. study [24], no evidence was found for any airway obstruction: none of the children with IBD showed abnormal FEV_1_/FVC or FEF_25–75_ values (>−1.96 z-scores). The mean FEV_1_/FVC value in the children with IBD was 0.96 (SD, ±0.80) z-scores. The authors stated that no screening was required for pulmonary manifestations in asymptomatic paediatric and adolescent patients with IBD.

We did not observe any differences related to the disease activity and the kind of therapy, including immunosuppressive therapy.

Contrary to the study by El Amrousy et al. [8], who revealed that spirometry results (FEV_1_, FVC, FEF 25% to 75%) obtained in children with newly diagnosed IBD, were not different from those in healthy children, but a significant deterioration was observed during the disease activity period. Furthermore, the patients at remission still had lower parameters than the normal controls. This study indicated that spirometry abnormalities were primarily dep6endent on the intestinal disease and its activity. 

### 5.2. FeNO

In our study, we tried to assess if FeNO results differed in the IBD children vs. those in the control group and to find out if the FeNO values could be used to identify IBD patients who would need further pulmonary evaluation. 

Since Koek et al. revealed [18] significant differences in FeNO values between adult patients with CD and UC and the controls, with the highest FeNO values identified in the UC group, a lot of studies have been trying to evaluate FeNO values as an indicator of pulmonary involvement in IBD. The results obtained so far have been contradictory though. 

The mean value of FeNO in our study group was not different from that in the healthy children (16.37 ± 11.46 ppb vs. 18.15 ± 12.71 ppb, respectively), as well as between CD and UC patients (16.06 ± 4.26 ppb vs. 16.64 ± 8.04 ppb, respectively). This is contrary to a Polish study [9], where children with ulcerative colitis had higher values of FeNO compared to a control group with the mean FeNO values (9.3 ± 3.3, 27.7 ± 14.8 and 16.6 ± 9.28 ppb in the CD, UC and the control group, respectively) (*p* < 0.0001). However only in four patients with UCdid the FeNO level exceed the upper limit of normal (>35 ppm). The UC patients presented significantly higher levels than the CD patients, which was explained by possible effects of the steroid therapy, applied in one CU patient.

Regarding the adult population, Ozyilmaz et al. also showed a difference in FeNO concentrations between IBD patients with and without pulmonary involvement (the mean values of 32 and 24 ppb, respectively); however, the mean values in both groups were within their normal limits. They concluded that a sensitivity and specificity analysis failed to determine the cut off-values for the FeNO levels, i.e., which could have reliably distinguished patients with and without pulmonary involvement [17]. Similarly to our study, Ikonomi et al. observed that IBD subjects had nearly the same FeNO levels as those without IBD (17.0 ± 16.2 vs. 16.7 ± 14.5 ppb, respectively). In their opinion, the measurements of FeNO values did not appear to be useful, either for IBD screening or for disease activity assessments [3]. 

In their latest paper, Ai et al. [25] discovered that, among 481 healthy children, the geometric mean value of FeNO was 14.59 ppb (the minimum and the maximum value was 5 and 69 ppb, respectively), and the upper limit for a normal value was 28 ppb, which could suggest inflammation in the airways. However, certain influencing factors, such as the age, body weight and height, should be considered as they correlate with FeNO results. In our study, no significant differences in FeNO values were found between the children with or without immunosuppressive treatment (*p* = 0.78). Furlano et al. [24] reported a similar observation. 

We also did not observe any differences in FeNO values in different stages of disease activity. This may have been because of several limitations such as there being a small sample size or the difficulty in determining the exact disease activity in the patients with immunosuppressive treatment. Corticosteroids potentially inhibit the expression of the inducible NO synthase pathway, leading to a decrease in the concentration of exhaled NO in the airways and, therefore, in FENO levels. Based on these limited and conflicting data, the FeNO results do not seem to be a reliable parameter for the identification of latent pulmonary involvement in paediatric patients with IBD.

### 5.3. Oscillometry

IOS is one of the techniques, defined by Dubois et al. 50 years ago, that allows for passive measurements of lung mechanics. There have been many studies showing that IOS is an effective method to evaluate airway pathologies in small children, especially in those with asthma, being useful both for diagnosis and disease monitoring [26]. The recent study by Kilci at al. demonstrated that the R (R5%, R10%, R5–20) and Ax values of IOS in patients in the nephrotic phase were higher than in the patients at remission and in controls. The authors concluded that IOS could be a reliable tool in children to evaluate respiratory function and it may even be superior to spirometry in small children [27]. To the best of our knowledge, no studies have yet been undertaken to investigate the respiratory function in IBD children by means of oscillometry. We demonstrated that, in the study group, the X5 z-score values were significantly lower than those in the control group (−0.01 ± 0.67 vs. 0.27 ± 0.63, respectively) and Fres was significantly higher (16.21 ± 4.2 vs. 14.16 ± 5.24, respectively.), which could have indicated a slightly increased airway resistance that reflected a small airway airflow limitation. Our observation was similar to those in other studies. Oppenheimer et al. [28] found that IOS was a non-invasive tool for the assessment of distal airway function when spirometry was normal, which can be applied to various clinical settings including the early diagnosis of chronic obstructive pulmonary disease. Su Gang-Gong et al. [29] also revealed that, in patients with chronic pulmonary disease, X5 was statistically lower than in the control group (R5, R20 and Fres were significantly higher) and MEF 50–75 differed significantly also from the values in the control group. In their opinion, the results indicated that, regarding the patients with COPD at stage 0, the pulmonary function was only relatively normal, as being opposed to absolutely normal.

Contradictory results may be found in many studies regarding pulmonary function abnormalities in paediatric IBD patients. The explanation for these divergences should be sought in different sample sizes, different genetics, the effects of different IBD therapies on PFT results, different age groups involved, different methods of PFTs used in paediatric patients, different times of PFT estimations, either in active disease or in its remission, and in the different durations and severities of the disease.

The limitations of our study included: it being a single center study, a low number of patients with active disease, and the lack of oscillometry tests in all the patients due to their conscious refusal. 

The strengths of our study were the following: it was a homogenous group of IBD patients, different lung tests were performed to cover the whole range of dysfunctions based on a possible respiratory involvement in IBD patients, the comparison of IBD patients with healthy controls to identify differences and the use of oscillometry as a complementary, pulmonary function assessing method. 

Summing up, our study demonstrated that spirometry results, obtained in the study group, differed from those in the healthy children, although they were still within their normal limits. In our opinion, it could be an indication to repeat the tests in the future. The oscillometry results showed slightly increased airway resistance. The mean FeNO levels, measured in the study group, did not differ from the values in the control group. The pulmonary function abnormalities did not depend on either immunosuppressive therapy or on disease activity. Thus, general screening of asymptomatic patients would appear to be a necessary measure, demanding suitable revisions in screening recommendations in this population.

## 6. Conclusions

Pulmonary function in IBD children differed from the control group in spirometric and oscillometric assessment. The pulmonary function abnormalities did not depend on either disease activity or on immunosuppressive therapy. FeNO values did not appear to be useful, either for screening IBD children for pulmonary involvement or to assess disease activity.

## Figures and Tables

**Table 1:** The parameters most frequently used in oscillometric examination.

Parameter	Description	Interpretation
R5	Respiratory system resistance at 5 Hz	R5 represents the total resistance of the respiratory system (extrathoracic, central or peripheral airways resistance)
R20	Respiratory system resistance at 20 Hz	R20 represents the resistance of the large airways
R5–20	Difference of resistances between 5–20 Hz	R5–R20 reflects the resistance of the peripheral airways
Fres	Resonant frequency	The frequency in which the respiratory system reactance equals 0
X5	Respiratory system reactance at 5 Hz	X5 represents the elastic recoil of the peripheral airways
X20	Respiratory system reactance at 20 Hz	X20 represents the elastic recoil of the large airways
Ax	Area of reactance	The sum of reactance values below resonance frequency were measured

R—resistance; X—reactance; Ax—area of reactance; Fres—Resonant frequency.

**Table 2:** Main changes in IOS parameters in respiratory disorders.

	R5	R20	X5	Fres	R5–R20
Distal obstruction	↑↑↑	normal or ↑	more normal	↑	R5 > R20
Proximal obstruction	↑↑	↑↑	Norma	Normal	R5 = R20
Restriction	Normal	Normal	more negative	↑↑	Normal

R—resistance; X—reactance; Fres—Resonant frequency.

**Table 3:** The characteristic features of the study group.

Value	All IBD Patients *n* = 72	*p*-Value *	CD *n* = 39	*p*-Value *	UC *n* = 33	*p*-Value *	Control Group *n* = 40
Mean age in years ± SD	14.45 ± 2.27	0.5918	14.77 ± 2.37	0.3392	14.18 ± 2.72	0.9812	14.17 ± 2.82
Female/male	36/36		10/29		23/10		19/21
Weight SDS	−0.32 ± 0.95	0.0019	−0.49 ± 0.93	0.0002	−0.17 ± 0.93	0.042	0.35 ± 1.37
Height SDS	−0.37 ± 1.12	0.4041	−0.6 ± 1.14	0.8991	−0.18 ± 1.08	0.1397	−0.56 ± 1.19
BMI SDS	−0.32 ± 0.87	0.0866	−0.35 ± 0.95	0.2278	−0.30 ± 0.81	0.1185	−0.67 ± 1.20
Mean duration of disease in years ± SD	2.02 ± 2.57		2.25 ± 2.27		1.83 ± 1.15		NA
Type of treatment:							
Immunosuppressive therapy	43		23		20		NA
Without immunosuppressive therapy	29		6		23		NA
Disease activity (the number of patients):							
Remission/Mild	57		34		23		NA
Moderate	10		4		6		NA
Severe	5		2		3		NA

IBD—inflammatory bowel disease; CD—Crohn’s disease; UC—ulcerative colitis; SDS—standard deviation score; BMI—body mass index; * *p*-Value—for comparison with the control group; NA—not applicable.

**Table 4:** A comparison of spirometry parameters, IOS and FeNO among the patients with CD, UC and the controls.

Value	All IBD Patients Mean (SD) *n* = 72	*p*-Value *	CD Patients Mean (SD) *n* = 39	*p*-Value *	UC Patients Mean (SD) *n* = 33	*p*-Value *	Control Group Number Mean (SD) *n* = 40
FEV_1_ z-score	−0.59 ± 1.08	0	−0.84 ± 1.10	0	−0.43 ± 1.08	0	−0.01 ± 0.85
FEV_1_/FVC z-score	−0.62 ± 1.1	0.0042	−0.83 ± 1.08	0.0005	−0.40 ± 1.06	0.0803	0.57 ± 1.02
FVC z-score	−0.26 ± 1.14	0.0002	−0.37 ± 1.18	0.0004	−0.17 ± 1.1	0.0028	0.54 ± 0.96
MEF_75_ z-score	−1.71 ± 1.51	0	−1.62 ± 1.65	0	−1.79 ± 1.41	0	−0.38 ± 0.97
MEF_50_ z-score	−0.30 ± 0.96	0	−0.53 ± 0.95	0	−0.12 ± 0.93	0.0032	0.50 ± 0.90
MEF_25_ z-score	−0.18 ± 0.56	0.0025	−0.43 ± 0.87	0.0003	0.02 ± 0.77	0.0982	0.34 ± 0.89
FeNO median	16.37 ± 11.46		16.06 ± 4.26		16.64 ± 8.040		18.15 ± 12.71
**Value**	***n* = 46**		***n* = 20**		***n* = 26**		***n* = 40**
R5 z-score	−0.13 ± 0.70	0.1457	−0.05 ± 0.75	0.1163	−0.19 ± 0.66	0.3706	−0.34 ± 0.42
R20 z-score	−0.28 ± 0.6	0.36	−0.32 ± 0.64	0.6183	−0.25 ± 0.59	0.3023	−0.39 ± 0.48
R(5–20) kPa/L/s	0.09 ± 0.09	0.6503	0.11 ± 0.11	0.3391	0.08 ± 0.07	0.8121	0.08 ± 0.09
X5 HZ z-score	−0.01 ± 0.67	0.0309	−0.06 ± 0.76	0.0687	0.01 ± 0.59	0.1053	0.27 ± 0.63
X20 z-score	−0.71 ± 0.62	0.1105	−0.76 ± 0.56	0.1322	−0.67 ± 0.68	0.2878	−0.47 ± 0.78
Ax	0.74 ± 0.61	0.0389	0.90 ± 0.67	0.1499	0.61 ± 0.54	0.885	0.63 ± 0.68
Resonant frequency (Fres)	16.21 ± 4.2	0.0457	17.13 ± 3.92	0.0266	15.46 ± 4.21	0.385	14.16 ± 5.24

IBD—inflammatory bowel disease; CD—Crohn’s disease; UC—ulcerative colitis; FEV1—forced expiratory volume in 1 s; FVC—forced vital capacity; MEF 75, 50, 25—maximal expiratory flow at 75, 50, 25 percent of vital capacity (respectively); %—percentage of predicted; AX—area of reactance; IOS—impulse oscillometry; R—resistance; X—reactance; Ax—area of reactance; * *p*-Value—for comparison with the control group.

**Table 5:** The clinical profile of the patients with or without immunosuppressive therapy.

Parameters	Immunosuppressive Therapy *n* = 43	Without Immunosuppressive Therapy *n* = 29	*p*-Value
Age (median ± SD)	14.61 ± 2.28	14.22 ± 2.96	NS
Sex (F/M)	22/21	14/15	NS
Mean of duration of the disease (year)	2.12 ± 3.20	1.88 ± 2.10	NS
Weight SDS	−0.47 ± 0.89	−0.09 ± 1.0	NS
Height SDS	−0.64 ± 1.0	0.03 ± 1.14	*p* = 0.01
BMI SDS	−0.36 ± 0.9	−0.27 ± 1.15	NS
Abnormal spirometry results, *n* (%)	32	17	
Obstructive pattern, *n* (%)	18	6.8	
Obstructive and small airway pattern, *n* (%)	4.6	3.4	
Restrictive pattern, *n* (%)	9.3	6.8	
FEV1% z-score	−0.61 ± 1.12	−0.52 ± 1.1	NS
FEV1/FVC z-score	−0.51 ± 1.1	−0.73 ± 1.06	NS
FVC z-score	−0.30 ± 1.26	−0.20 ± 0.93	NS
MEF_25_ z-score	−0.18 ± 0.95	−0.18 ± 0.67	NS
MEF_50_ z-score	−0.31 ± 0.96	−0.30 ± 1.01	NS
MEF_75_ z-score	−1.65 ± 1.5	−1.81 ± 1.08	NS
FeNO (ppb)	16.06 ± 13.27	16.82 ± 8.29	NS
R5 z-score	−0.09 ± 0.72	−0.20 ± 0.67	NS
R20 z-score	−0.23 ± 0.65	−0.39 ± 1.63	NS
X5-z-score	−0.06 ± 0.61	0.06 ± 1.65	NS
X20 z-score	−0.73 ± 0.59	−0.69 ± 0.70	NS
Ax	0.74 ± 0.54	0.75 ± 0.68	NS
R5–20%	0.10 ± 0.08	0.09 ± 0.11	NS
Resonant frequency (Fres)	16.9 ± 4.32	16.44 ± 3.86	NS

FEV1—forced expiratory volume in 1 s; FVC—forced vital capacity; MEF 75, 50, 25—maximal expiratory flow at 75, 50, 25 percent of vital capacity (respectively); %, percentage of predicted; AX, area of reactance; R—resistance; X—reactance; Ax—area of reactance, NS—Not significant; *p*-values for overall differences between groups.
